# The *LmSNF1* Gene Is Required for Pathogenicity in the Canola Blackleg Pathogen *Leptosphaeria maculans*

**DOI:** 10.1371/journal.pone.0092503

**Published:** 2014-03-17

**Authors:** Jie Feng, Hui Zhang, Stephen E. Strelkov, Sheau-Fang Hwang

**Affiliations:** 1 Crop Diversification Centre North, Alberta Agriculture and Rural Development, Edmonton, Alberta, Canada; 2 The Institute of Vegetables and Flowers, Chinese Academy of Agricultural Sciences, Beijing, China; 3 Department of Agricultural, Food and Nutritional Science, University of Alberta, Edmonton, Alberta, Canada; Seoul National University, Republic of Korea

## Abstract

*Leptosphaeria maculans* is a fungal pathogen causing blackleg in canola. Its virulence has been attributed, among other factors, to the activity of hydrolytic cell wall degrading enzymes (CWDEs). Studies on the pathogenicity function of CWDEs in plant pathogenic fungi have been difficult due to gene redundancy. In microorganisms many CWDE genes are repressed by glucose and derepressed by the function of the sucrose non-fermenting protein kinase 1 gene (*SNF1*). To address the molecular function of *SNF1* in *L. maculans*, the ortholog of *SNF1* (*LmSNF1*) was cloned and functionally characterized using a gene knockout strategy. Growth of the *LmSNF1* knockout strains was severely disrupted, as was sporulation, spore germination and the ability to attach on the plant surface. When inoculated on canola cotyledons, the *LmSNF1* knockout strains could not cause any symptoms, indicating the loss of pathogenicity. The expression of 11 selected CWDE genes and a pathogenicity gene (*LopB*) was significantly down-regulated in the *LmSNF1* knockout strains. In conclusion, knockout of *LmSNF1* prevents *L. maculans* from properly derepressing the production of CWDEs, compromises the utilization of certain carbon sources, and impairs fungal pathogenicity on canola.

## Introduction

Plant pathogenic fungi secrete an array of cell wall degrading enzymes (CWDEs) capable of depolymerizing the polysaccharides of primary cell walls [1]–[3]. These enzymes received special attention from researchers and many of them have been demonstrated to be important for the pathogenicity of various fungi. However, as a result of the redundancy of genes and enzymatic activity, reverse genetic approaches that rely on single gene deletion or silencing have not effectively addressed the importance and function of these enzymes in pathogenicity [4]–[7].

In many microorganisms including fungi, CWDEs are subject to catabolite repression, a mechanism that controls the preferential use of easily fermentable carbon sources, such as glucose, by repressing genes that are used to metabolize other carbon sources, such as sucrose, galactose, pectin, and xylose [8], [9]. To test the role of CWDEs as a whole in fungal pathogenicity, disruption of the elements that control the derepression mechanism could be a more reliable approach than working on single CWDE genes [10]. In *Saccharomyces cerevisiae*, the *SNF1* (sucrose non-fermenting 1) gene has been shown to play a central role in carbon catabolite repression [11]. *SNF1* encodes a protein kinase (Snf1) that phosphorylates Mig1, a DNA-binding transcriptional repressor responsible for catabolite repression [12], leading to the derepression of all genes under Mig1 control. The ortholog of Mig1 in filamentous fungi is called creA [8]. Repression of CWDE genes by creA has been studied in a number of plant pathogenic fungi [13]–[16].

*SNF1* orthologs have been studied in plant pathogenic fungi, including *Cochliobolus carbonum* [10], *Fusarium oxysporum* [17], *Sclerotinia sclerotiorum* [18], *Magnaporthe oryzae* [19], *F. graminearum* [20], *Alternaria brassicicola* [13], *Verticillium dahliae* [21] and *Penicillium digitatum* [22]. In most of these studies, disruption of *SNF1* resulted in a reduction in the ability of the corresponding species to grow on certain carbon sources and in transcription of many CWDE genes. Components of fungal pathogenicity such as sporulation, spore germination and appressorium formation were impaired in the gene knockout strains and, consequently, the virulence on the host plants was reduced.

Blackleg of canola (*Brassica napus*), caused by the Dothideomycete *Leptosphaeria maculans* and *L. biglobosa*, is an important disease in many countries [23], [24] including Canada, Australia and most of Europe. Compared to *L. biglobosa*, *L. maculans* is more aggressive [25]. Worldwide, the two species coexist in many regions, with *L. maculans* typically causing cortical infection near the base of the stem and *L. biglobosa* resulting in more superficial stem lesions or pith damage [26]. In China, although blackleg is prevalent, it is caused only by *L. biglobosa* [25]. In western Canada, both species may be found in association with basal-stem canker symptoms, with *L. biglobosa* identified at lower frequencies [27].

In spite of the economic importance of blackleg disease, the pathogens involved have not been studied in great detail at the molecular level until recently. Four avirulence genes, *AvrLm1* [28], *AvrLm6* [29], *AvrLm4–7* [30] and *AvrLmJ1* [31] have been cloned and characterized. A few protein-encoding genes playing a role during pathogenesis also have been functionally studied, including an isocitrate lyase gene [32], the *lopB* gene with unknown function [33], the *THIOL* gene encoding a 3-Ketoacyl-CoA thiolase [34], the *Ipa* gene [35], the *Lmpma1* gene encoding a plasma membrane H^+^-ATPase isoform [36], the *Lmgpi15* gene encoding a component of the glycosylphosphatidylinositol anchor biosynthesis pathway [37], the *LmIFRD* gene [38], and the *Lmepi* gene encoding a key enzyme of the Leloir pathway [39]. In most of these reports, gene disruption mutants were studied and the disruption was achieved exclusively by the method of *Agrobacterium tumefaciens*-mediated random insertional mutagenesis.

The recent release of the whole genome sequence of *L. maculans* [40] laid a foundation for genomic studies of the blackleg pathosystem. While global studies of genes involved in this host-pathogen interaction have become easier, functional studies of pathogenicity genes by targeted gene knockout also are much facilitated. However, it is has been reported that in *L. maculans* at least 7 kb of flanking DNA is required for homologous recombination, an innate mechanism on which targeted gene knockout relies, and up to several hundred transformants need to be screened to achieve gene replacement [41]. To date, characterization of pathogenicity genes by targeted gene knockout has only been reported once in this fungal species [42]. Thus in the present study, we report results from a targeted gene knockout of the *L. maculans SNF1* gene and the subsequent characterization of the gene knockout stains. We demonstrate that *SNF1* is required for expression of genes involved in cell wall degradation and for spore germination, spore attachment and pathogenicity on canola plants.

## Materials and Methods

### Ethics statement

No specific permission was required for the field from which the *L. maculans* wild-type was derived. All of the field studies were carried out in a closed and protected green house or a growth chamber in Crop Diversification Centre North. This study did not involve endangered or protected species.

### Chemicals and standard techniques

All chemicals were purchased from Fisher Scientific (Ottawa, ON, Canada) unless otherwise specified. Polymerase chain reactions (PCR) were conducted in an Eppendorf Mastercycler thermal cycler (Eppendorf, Hamburg, Germany) using the PCR master mix from Promega (Madison, WI, USA). Quantitative PCR (qPCR) assays were conducted in a StepOnePlus Real-Time PCR System (Life Technologies, Carlsbad, CA, USA) using the GoTaq 2-Step RT-qPCR System (Promega) or the Power SYBR Green PCR Master Mix (Life Technologies). qPCR primers were designed with the online program Primer3web (http://primer3.ut.ee). DNA purification of amplicons from the reaction mixture or agarose gel was performed using a Wizard SV Gel and PCR Clean-Up System (Promega). Similarity searches of nucleic acid and protein sequences were performed using the Basic Local Alignment Search Tool (BLAST) against the National Center for Biotechnology Information (NCBI) database unless otherwise specified. Sequencing was done by the Department of Biological Sciences, University of Alberta (Edmonton, AB, Canada). Other molecular techniques, if not specified, were performed according to the protocols described by Sambrook and Russell [43].

### Sequence retrieving and analyses

**Figure 1:**
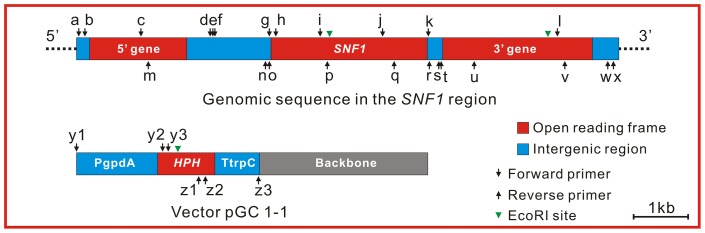
Diagram of the gene replacement constructs. 5′ gene and 3′ gene, the gene located on the 5′ upstream and 3′ downstream of *LmSNF1*; PgpdA, *Glomerella cingulata gpdA* promoter; *HPH*, *E. coli* hygromycin phosphotransferase gene; TtrpC, *Aspergillus nidulans trpC* terminator, a-z3, primers whose sequences can be identified in Table S1; The arrow heads indicate the EcoRI cutting sites.

Using the sequence of the open reading frame (ORF) of the *SNF1* gene from *C. carbonum* ([10]; GenBank accession number: AAD43341) as a query, a BLAST search was conducted against the *L. maculans* whole genome database (http://genome.jgi-psf.org/Lepmu1). This search produced a single hit which are referred to as *LmSNF1* hereafter in this report. The DNA sequence, including the ORF of *LmSNF1* and 3,500 bp of its 5′ upstream and 3′ downstream regions, was retrieved and served as the basis for the design of primers for PCR and qPCR analyses (Figure 1, Table S1). Three PCR fragments covering the ORF were amplified from fungal genomic DNA using the primer pairs g/p, i/q and j/r (Figure 1, Table S1) and sequenced using the same primers.

Orthologs of Snf1 from other fungi were identified by searching each of the accessible fungal genomes hosted at the Broad Institute (http://www.broadinstitute.org) Fungal Genome Database by BLAST with the deduced amino acid sequence of *LmSNF1* (referred to as LmSnf1) as the query. The amino acid sequence of the best hit from each fungal species was retrieved.

Twelve CWDE genes were selected from the *L. maculans* carbohydrate-active enzymes (CAZy) database (http://www.cazy.org/e1438.html). Three other protein-encoding genes that have been shown to be pathogenicity-related were selected from the pathogen host interactions (PHI) database (http://www.phi-base.org). Based on the mRNA sequence for each of these genes, a pair of qPCR primers was designed (Table S2).

Molecular weight and the isoelectric point (pI) of LmSnf1 were calculated using the online compute pI/Mw tool (http://web.expasy.org/compute_pi). Multiple alignments between LmSnf1 and its orthologs and the subsequent construction of a phylogenetic tree were performed with ClustalW2 (http://www.ebi.ac.uk/Tools/msa/clustalw2).

### Fungal and plant materials

A blackleg susceptible canola cultivar Westar was used as the host for *L. maculans.* The *L. maculans* strain S4, isolated from an infected canola plant in a field (GPS coordinates: 53.578, −113.222) in Strathcona County, Alberta, Canada, in 2012 was used as the wild-type and is referred to as WT hereafter. The WT and transformants were maintained on 4% potato dextrose agar (PDA) or V8 agar (150 ml V8 juice, 1.5 g CaCO_3_ and 15 g agar for 1 L) in Petri dishes. For cultures to be used in quantitative assays, 25 ml of medium was placed in each 10-cm diameter Petri dish with a peristaltic pump (Integra Biosciences, Hudson, NH, USA).

### Plant inoculation

Seeds of ‘Westar’ were sown in 50-ml plastic cups filled with Sunshine mix #4 soil (Sun Gro Horticulture, Vancouver, BC, Canada). The seedlings were maintained in a growth chamber with a 16 h photoperiod at 22°C day/18°C night. To prepare the inoculum, 14-day-old sporulating cultures of the fungus on V8 agar were flooded with sterile distilled water and the resultant conidial suspension was passed through eight layers of cheesecloth. Before inoculation, the paraxial surface of the cotyledons of 10-day old seedlings was wounded with a pair of forceps. The wounded sites were inoculated with a 5-µl droplet of the conidial suspension. After the inoculum was allowed to air dry, the plants were incubated in transparent plastic bags to maintain high relative humidity. After 48 hours, bags were removed and the plants were maintained in the growth chamber.

### Nucleic acid manipulations

Genomic DNA was extracted from fungal mycelia or infected plant tissue using a Qiagen DNeasy Plant Mini Kit (Qiagen, Toronto, ON, Canada) unless otherwise specified. Total RNA was isolated from frozen mycelium or inoculated plant tissue with an RNeasy Plant Mini Kit (Qiagen). The RNA samples were further treated with RNase-Free DNase (Qiagen) to remove DNA contaminants. The purified RNA was used for synthesis of the first stranded cDNA using an iScrip cDNA Synthesis Kit (Bio-Rad, Hercules, CA, USA) or a Promega GoTaq 2-Step RT-qPCR System (Promega).

### Transcript induction

For *in vitro* induction of transcripts, mycelium of WT generated after 48 h incubation in YPG broth (3 g yeast extract, 10 g peptone and 20 g glucose for 1 L) was transferred to fresh YPG broth, Czapek-Dox minimal medium (referred to as CD hereafter; 2 g NaNO3, 0.5 g KCl, 1 g K_2_HPO_4_, 0.5 g MgSO_4_·7H_2_O and 0.01 g FeSO_4_·7H_2_O for 1 L) or CD supplemented with 15% V8 juice or 1% glucose or pectin as the sole carbon source and incubated with agitation for another 24 h. The mycelia were harvested and subjected to RNA extraction as above.

For *in planta* induction, WT was inoculated on ‘Westar’ and samples were collected from inoculated cotyledons by excising the infection areas at 0, 12, 24, 48, 72, 96, 120 and 144 hours after inoculation (hai).

### Quantitative PCR

For qPCR analysis, the *L. maculans* housekeeping gene encoding *ACT1* (GenBank accession number: FJ172241) was used as an endogenous control and amplified with the primers ActinF: AGTGCGATGTCGATGTCAG and ActinR: AAGAGCGGTGATTTCCTTCT [38]. Primers for *LmSNF1* were the primer pair i/p (Figure 1, Table S1) and those for the selected CWDE and other pathogenicity-related genes are listed in Table S2.

The qPCR was conducted in a 15-µl reaction mixture containing 1.25 µl each of 5 µM forward and reverse primer and 200 ng of the cDNA template. The amplification conditions consisted of an initial denaturation step of 95°C for 10 min, followed by 40 cycles of 15 s at 95°C and 1 min at 60°C. After completion of the PCR amplification, a melting curve analysis was run to evaluate the amplification specificity. Primer efficiency was verified using standard curves generated from the wild-type genomic DNA. Expression of genes was calculated via the delta-delta method, with relative expression  = 2^− (ΔCt sample–ΔCt control)^ [44]. All qPCR amplifications were performed in triplicate with each of three cDNA replicates derived from different preparations of mycelium or plant tissue.

### Construction of transforming DNA for gene knockout

Two sets of transforming DNA were constructed by fusion PCR [45] and each set was used in fungal transformation. To generate the first construct, a 3.2 kb cassette containing the *Escherichia coli* hygromycin B phosphotransferase gene (*HPH*) that has been rendered suitable for fungal expression was amplified from the vector pGC1-1 [46] using the primer pair y1/z3 (Figure 1, Table S1). Two genomic fragments at 1.1 kb and 0.3 kb were amplified from the 5′ and 3′ flanking regions of *LmSNF1* using the primer pairs d/o and k/t, respectively. The 5′ ends of the primers o and k were tailed with the reverse-complemented sequences of primers y1 and z3, respectively. The three amplified fragments were mixed at a molar ratio of 1∶1∶1 and used as a template to amplify a 4.6 kb fusion fragment with the primer pair e/s. The resulting fragment was cloned into the pGEM T-Easy vector (Promega) by TA cloning.

For the second construct, two fusion fragments were generated by a split-marker strategy [47]. To generate the first fusion fragment, a 2.3 kb fragment was amplified from pGC1-1 using the primer pair y1/z2 and a 3.4 kb fragment was amplified from the 5′ flanking region of *LmSNF1* with the primer pair a/o. These two fragments were mixed at a molar ratio of 1∶1 and used as template to amplify a 5.7 kb fusion fragment with the primer pair b/z1. For the second fusion fragment, a 1.7 kb fragment was amplified from pGC1-1 using the primer pair y2/z3 and a 3.3 kb fragment was amplified from the 3′ region of *LmSNF1* using the primer pair k/x. Primer k was tailed at the 5′ end with the reverse-complemented sequence of primer z3. These two fragments were mixed at a molar ratio of 1∶1 and used as a template to amplify a 5.0 kb fusion fragment using the primer pair y3/w. The 5.7 and 5.0-kb fusion fragments were cloned into the pGEM T-Easy vector by TA cloning.

### Fungal transformation

PEG-mediated protoplast transformation followed the protocol of Feng et al. [48]. Each transformation experiment was conducted in triplicate in 1.5-ml tubes each containing 3×10^7^ protoplasts in a volume of 300 μl and supplemented with 30 μl of transforming DNA. The DNA consisted of 3 μg of the first construct or 2 μg of each of the two fragments of the second construct, all of which were generated by NotI digestion of the corresponding plasmid DNA followed by gel purification. The hygromycin B-resistant colonies that grew on the selective media were transferred to PDA plates containing 100 µg/ml of hygromycin B for a second round of screening. Colonies identified in the second round of screening were regarded as successful transformants. Unless otherwise specified, growth medium for the transformants was always supplemented with 100 µg/ml of hygromycin B.

### Confirmation of gene knockout

Genomic DNA of WT and all transformants was extracted from mycelium generated on PDA plates according to the method described by Feng et al. [49]. Based on these DNA samples, transformants were screened for a gene replacement event by PCR analysis using primers specific to *HPH*, the *LmSNF1* ORF or the genes located on the 5′ and 3′ regions of *LmSNF1*.

Based on the PCR results, all of the putative gene knockout (Ko) strains, one ectopic insertion strain (Ect) and WT were subjected to Southern analysis using a DIG DNA Labeling and Detection kit (Roche Diagnostics, Laval, QC, Canada). All strains were grown in YPG broth for two days and genomic DNA was extracted as per the phenol protocol [43]. Twenty µg of genomic DNA from each strain was digested with EcoRI and fractionated on a 1% agarose gel. A 1.7-kb fragment amplified from WT using the primer pair j/u was used as the probe.

Gene knockout events were further confirmed by qPCR analysis. Total RNA was extracted from healthy canola, WT, Ect and the six Ko strains after transcript induction in CD medium for 24 hours. Transcript accumulation of *LmSNF1* and its 5′ and 3′ genes was assessed.

### Growth assay

Growth of WT and the transformants was measured on Petri dishes containing CD medium supplemented with 15% V8 juice or 1% glucose or pectin as the sole carbon source (all media without hygromycin B). Briefly, culture plugs (0.5 cm in diameter) were excised from the margins of a colony growing on PDA and were transferred to the center of freshly-prepared plates. The plates were incubated under continuous light at 25°C and colony diameter was measured in two directions at right angles to each other after 6 days. The average colony radius was calculated and used to represent fungal growth. This experiment was conducted as a randomized complete block design with three replicates and repeated with similar results obtained.

To measure mycelial growth in liquid media, three plugs (0.5 cm in diameter) were inoculated into 50 ml of medium in a 125-ml flask. The media used were same as the plate assay but without agar. The flasks were shaken at 150 rpm at 25°C for 6 days. The mycelial mats were vacuum-filtered through two layers of filter paper and lyophilized. The mass of the lyophilized mycelium was recorded. This experiment was conducted as a randomized complete block design with three replicates and repeated with similar results obtained.

### Sporulation assay

The ability of the transformants to produce conidia was assessed by induction of sporulation on plates containing 25 ml V8-agar without hygromycin B under continuous light at 25°C. After 10 days, conidia formed on the plate were harvested in 100 ml H_2_O and filtered through eight layers of cheesecloth. The conidial concentration in the suspension was measured with a haemocytometer and expressed as the number of conidia per plate or per square millimeter of the V8-agar culture. This experiment was conducted as a randomized complete block design with three replicates and repeated with similar results obtained.

### Germination assay

Ten ml of 0.8% PDA at 45°C was mixed with 100 μl of conidial suspension at a concentration of 2×10^7^ conidia/ml. The mixture was poured into a Petri dish and kept at 25°C in the dark. After 24 hours, a small piece was excised and transferred to a haemocytometer and covered with a cover slide. The cover slide was pressed with one click of a retractable pilot pen to distribute the mixture evenly in the counting chamber. Under a Zeiss AXIO microscope (Carl Zeiss, Thornwood, NY, USA), the total number of conidia and number of germinated conidia were counted within an area of 0.36 mm^2^ (nine 0.2×0.2 mm cells) in the center of the counting chamber. For each strain, three plates (repeats) from different conidial suspensions were prepared and from each plate 10 samples were counted. This experiment was repeated with similar results obtained.

### Attachment assay

A conidial suspension from each strain was prepared and adjusted to a concentration of 1×10^7^ conidia/ml. Two 10-μl droplets of the suspension were applied to the surface of a cotyledon excised from a 10-day old ‘Westar’ seedling. The cotyledons were placed on water-soaked filter paper in Petri dishes for two hours to let the droplets dry. The Petri dishes were sealed and kept in darkness at room temperature. At 6 and 20 hai, the attachment of conidia to the surface of the cotyledon was assessed by counting the number of washed-off conidia after washing. Briefly, one inoculated cotyledon was transferred into a 2-ml tube containing 1 ml washing buffer (0.02% Tween 20). The tubes were shaken at 1,400 rpm for 30 seconds with an Eppendorf Thermomixer. An 800 μl aliquot of the suspension was transferred into a 1.5 ml tube. After centrifugation at 14,000 rpm for 2 min, 700 μl of the supernatant was removed and the remaining 100 μl was used to quantify the spores. For each sample, two counts were made in a 0.04 mm^2^ area (one 0.2×0.2 mm cell at the center of the counting chamber) and 10 samples were examined for each tube. The average of the 20 counts was used to calculate the attachment ratio, which was regarded as the data from one experiment unit. For each strain, the attachment was assessed on three cotyledons (repeats).

### Pathogenicity test

Inoculation of ‘Westar’ was conducted as described above. On each cotyledon, inoculation of a Ko or the Ect strain was accompanied by inoculation with WT side by side. For each strain, 50 cotyledons on approximately 30 plants in five pots were inoculated with conidial suspension at a concentration of 1×10^6^ conidia/ml or 1×10^7^ conidia/ml. Plants were examined daily from 4 to 10 days after inoculation (dai) for the development of blackleg symptoms.

### Statistical analysis

Data were analyzed for statistical significance using the general linear model (GLM) procedure of the Statistical Analysis System (SAS Institute, Cary, NC, USA). All of the data were subjected to one-way analysis of variance and, when appropriate, a multiple-range test was conducted by Fisher's least significant difference (LSD) at *P*≤0.01.

## Results

### *LmSNF1* shares high sequence similarity with its orthologs

Sequencing of the *LmSNF1* PCR fragments revealed an ORF of 2,782 bp, which is identical to the *SNF1* gene (XM_003844721) in the whole-genome sequenced *L. maculans* strain JN3. The ORF is interrupted by two introns of 59 and 53 bp. It encodes a polypeptide of 889 amino acids with a calculated molecular weight of 99 kDa and a pI of 8.8.

LmSnf1 shares 82%, 80% and 79% overall identities with its orthologs in *Stagonospora nodorum* (AAR02440), *C. carbonum* (AAD43341) and *Pyrenophora tritici-repentis* (XP_001932670), respectively. Multiple alignment of LmSnf1 with its orthologs in other fungi indicated that their sequence homology is higher in the N-terminal kinase domain and lower in the C-terminal regulatory domain (data not shown). A phylogenetic tree was constructed based on the alignment. Comparison of this tree with the phylogenomic tree illustrated at the Broad Institute Fungal Genome database, which was constructed using more than 70 genes that are conserved among all fungal species (Dr. Li-Jun Ma, Broad Institute, personal communication), revealed a high consistence between the two trees, especially among the species in Ascomycota (Figure S1).

### Expression of *LmSNF1* is induced by pectin and during pathogenesis

**Figure 2:**
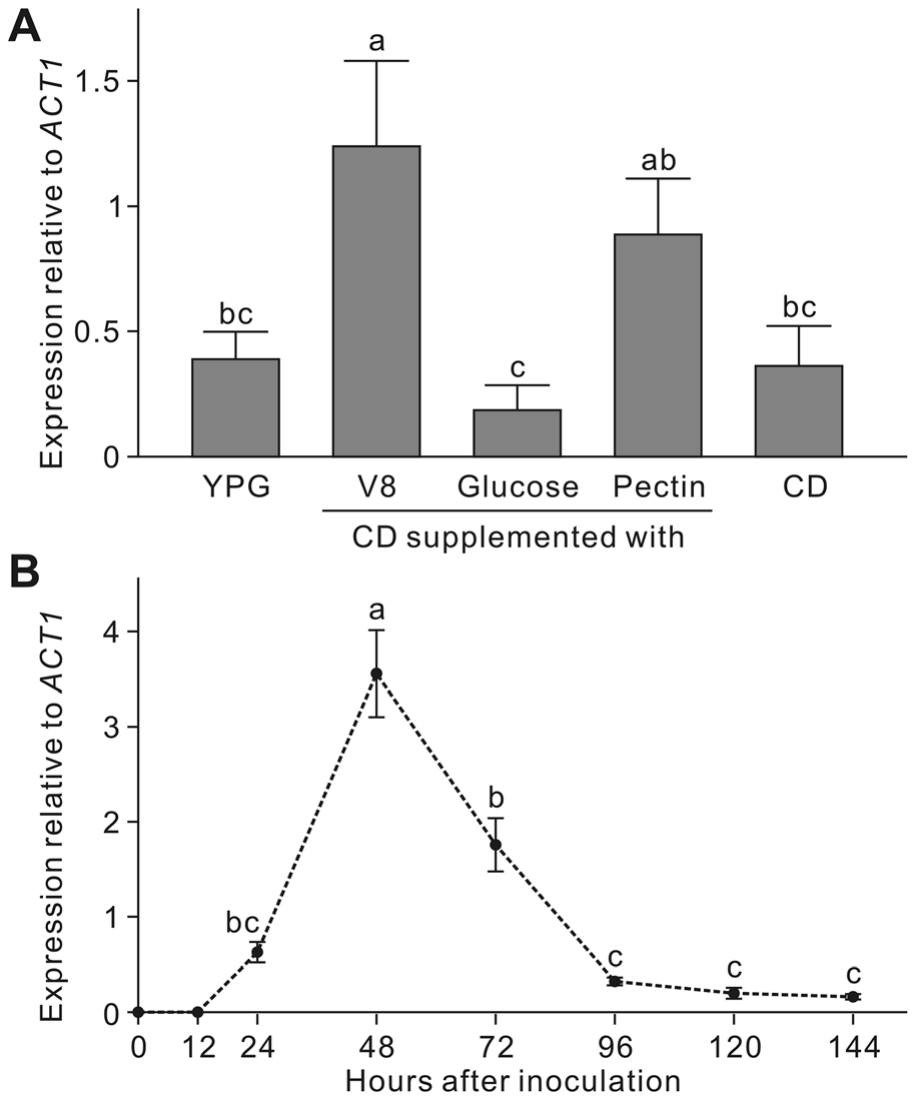
Expression analysis of *LmSNF1* in the wild type of *Leptosphaeria maculans* by quantitative PCR. **A,** Expression of *LmSNF1* 24 h after culturing in YPG or minimal media (CD) supplemented with various carbon sources. **B,** Expression of *LmSNF1* in infected canola tissue. Canola cultivar Westar was inoculated with the fungus and the infected plant samples were collected at 0–144 hours after inoculation. Means in the plot topped by the same letter do not differ based on Fisher's LSD test at *P*≤0.01 (n = 3).

In YPG, CD and CD supplemented with glucose, expression of *LmSNF1* was at lower levels than the housekeeping gene *ACT1*. Expression of *LmSNF1* increased in V8 and pectin media, in which it was similar to *ACT1*. Compared to the glucose medium, expression in V8 and pectin media was significantly higher (Figure 2A).

*In planta* expression of *LmSNF1* was investigated over a 0–144 hai time course. No transcript of either *LmSNF1* or *ACT1* could be detected at 0 and 12 hai. From 24 to 96 hai, expression of *LmSNF1* increased and then decreased, with an expression peak at 48 hai. From 96 to 144 hai, expression remained constant but at a relative low level (Figure 2B). This result indicates that the activation of *LmSNF1* expression is concurrent with an early stage of infection by the fungus.

### *LmSNF1* was knocked out from the fungal genome

Transformations using the first and second constructs produced 21 and 5 transformants, respectively. PCR screening for the absence of *LmSNF1* revealed that 2 out of 21 strains transformed with the first construct and four out of five strains transformed with the second construct were putative gene knockout strains (Figure S2). These strains were named Ko1 and Ko2 (with the first construct) and Ko3 to Ko6 (with the second construct). The ectopic insertion strain (Ect) transformed with the second construct, in which *HPH* was integrated into the genome but *LmSNF1* remained unchanged, was used as a control in addition to WT.

**Figure 3:**
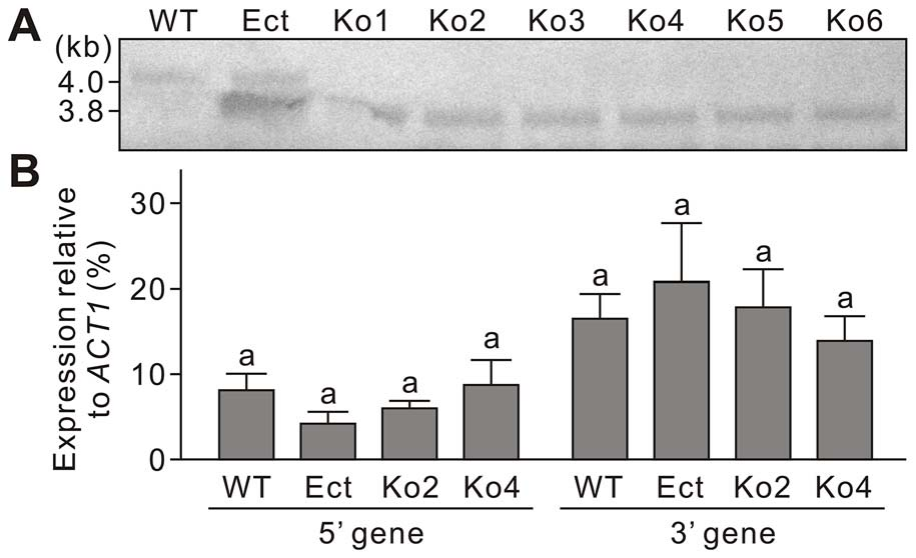
Analysis of *LmSNF1* gene knockout mutants of *Leptosphaeria maculans*. WT, the wild-type; Ect, the ectopic insertion strain; Ko, *LmSNF1* knockout strains. **A,** Southern blot. Genomic DNA from the fungal strains was digested with EcoRI and hybridized with a PCR fragment that encompasses a part of *LmSNF1* open reading frame and its 3′ region. **B,** Assessment of expression of the 5′ and 3′ genes by quantitative PCR. Means in the plot topped by the same letter do not differ based on Fisher's LSD test at *P*≤0.01 (n = 3).

The gene knockout event was confirmed by Southern (Figure 3A) and qPCR analyses. A hybridization signal was observed in WT at 4.0 kb, which is the position of the band produced by digestion with EcoRI at two sites, one within the *LmSNF1* ORF and another at the 3′ region (Figure 1). In Ko1-Ko6, signals were detected at 3.8 kb, which would have resulted from the same cutting site in the 3′ region and another cutting site within the *HPH* ORF (Figure 1). From Ect, hybridization signals were observed at both 4.0 and 3.8 kb, indicating the presence of both *HPH* construct and *LmSNF1*. Against the cDNA, amplification of *LmSNF1* could be detected by qPCR analysis from WT and Ect, but not from the Ko strains. These results indicate that the six strains (Ko1-6) are true gene knockout strains.

To confirm that knockout of *LmSNF1* did not affect its 5′ and 3′ genes, a qPCR analysis was conducted with cDNA from YPG cultures of WT, Ect, Ko2 and Ko4. No significant differences in the expression of either the 5′ or the 3′ gene could be detected among these four strains (Figure 3B).

### Knockout of *LmSNF1* affects vegetative growth and sporulation

After 6 days of incubation on CD supplemented with V8 juice, glucose or pectin, all of the six gene knockout strains exhibited slower growth relative to WT and Ect (Table 1). This became even more evident after 11 days of incubation (Figure S3). Similarly, in all of the three liquid media, the Ko strains produced less dry weight than WT and Ect after 6 days of incubation (Table 1). These results indicate that the vegetative growth of *L. maculans* is impaired as a result of the loss of *LmSNF1*.

**Table 1:** Growth of the *Leptosphaeria maculans* wild-type, one ectopic insertion strain and six *LmSNF1* knockout strains on solid and in liquid media.

Strain	Radius on plates (mm)			Dry weight in liquid media (g)		
	V8	Glucose	Pectin	V8	Glucose	Pectin
WT	29.5±0.6 a^a^	25.8±0.8 a	20.1±1.5 a	0.37±0.01 a	0.18±0.01 a	0.17±0.01 a
Ect	29.7±0.4 a	27.3±1.2 a	14.1±1.2 ab	0.31±0.01 ab	0.18±0.02 a	0.15±0.01 ab
Ko1	11.9±0.2 b	8.7±0.9 b	5.9±1.3 c	0.20±0.02 c	0.06±0.01 b	0.09±0.01 b
Ko2	10.8±0.9 b	11.1±0.5 b	11.6±1.1 b	0.29±0.01 b	0.08±0.01 b	0.15±0.01 ab
Ko3	11.6±0.4 b	11.5±0.3 b	6.5±1.2 bc	0.24±0.03 bc	0.08±0.01 b	0.12±0.02 ab
Ko4	13.2±0.2 b	8.9±0.4 b	7.7±0.9 bc	0.18±0.02 c	0.05±0.02 b	0.10±0.01 ab
Ko5	11.1±0.5 b	10.2±0.6 b	8.1±0.3 bc	0.23±0.02 bc	0.07±0.01 b	0.12±0.02 ab
Ko6	13.2±0.5 b	11.7±0.3 b	10.0±1.3 bc	0.25±0.01 bc	0.10±0.01 b	0.12±0.02 ab

a Data (mean±SE) followed by the same letter do not differ based on Fisher's LSD test at *P*≤0.01 (n = 3).

**Figure 4:**
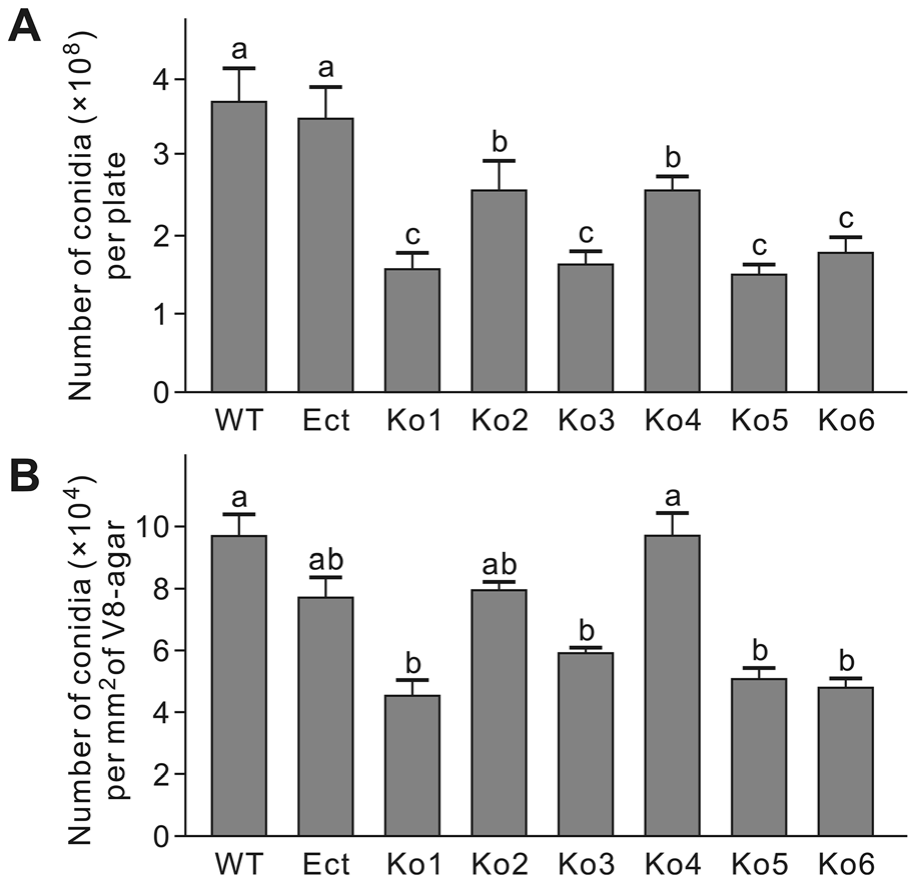
Sporulation of *Leptosphaeria maculans* strains on V8 agar plates assessed by number of conidia per plate (A) and number of conidia per mm^2^ of culture (B). WT, the wild-type; Ect, the ectopic insertion strain; Ko, *LmSNF1* knockout strains. Means in the plot topped by the same letter do not differ based on Fisher's LSD test at *P*≤0.01 (n = 3).

After 10 days of growth on V8-agar, abundant sporulation was evident for all strains. Assessment of the number of conidia per plate indicated that all Ko strains produced less conidia than WT and Ect. Among the Ko strains, Ko2 and Ko4 produced the most conidia (Figure 4A). When sporulation was assessed as the number of conidia per area of colony, Ko1, Ko3, Ko5 and Ko6 produced fewer conidia than WT, while Ko2 and Ko4 did not exhibit a significant decrease in sporulation (Figure 4B). These data suggest that, at least in the strains Ko1, Ko3, Ko5 and Ko6, knockout of *LmSNF1* impairs fungal sporulation.

### Knockout of *LmSNF1* affects conidial germination and attachment on leaf surface

**Figure 5:**
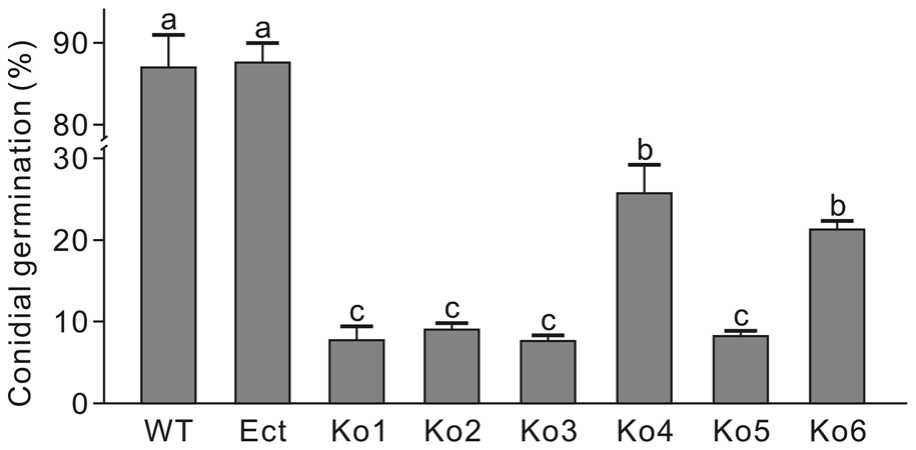
Conidial germination rate of *Leptosphaeria maculans* strains. Before counting the germinated and the total number of conidia under a microscope, conidia were incubated in 0.8% potato dextrose agar for 24 hours. WT, the wild-type; Ect, the ectopic insertion strain; Ko, *LmSNF1* knockout strains. Means in the plot topped by the same letter do not differ based on Fisher's LSD test at *P*≤0.01 (n = 3).

Conidial germination of all strains was investigated in 0.8% PDA (Figure S4). After 24 hours, 87.7% and 88.2% of conidia from WT and Ect, respectively, had germinated, percentages which were significantly higher than for conidia of the six Ko stains. Within the Ko strains, Ko4 and Ko6 showed a higher germination rate than the others (Figure 5).

**Figure 6:**
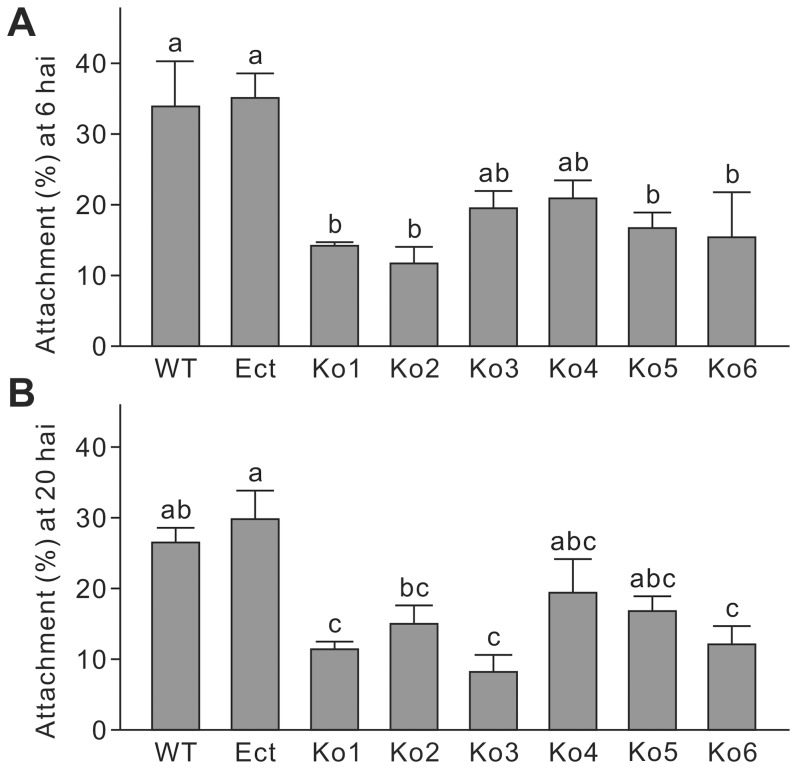
Conidial attachment on canola cotyledons of *Leptosphaeria maculans* strains. WT, the wild-type; Ect, the ectopic insertion strain; Ko, *LmSNF1* knockout strains. Two 10-μl droplets of a spore suspension (1×10^7^ conidia/ml) were applied on the surface of one cotyledon. At 6 (**A**) and 20 (**B**) hours after inoculation, the cotyledon was washed with water containing 0.02% Tween 20. The washed out spores were counted and the ratio of attachment was calculated. Means in the plot topped by the same letter do not differ based on Fisher's LSD test at *P*≤0.01 (n = 3).

Conidial attachment to the plant surface was assessed at 6 and 20 hai. Compared to WT and Ect, the strains Ko1, Ko2, Ko5 and Ko6 showed significantly impaired conidial attachment rates at 6 hai (Figure 6A). At 20 hai, Ko1, Ko3 and Ko6 showed reduced attachment (Figure 6B). Although the reduction in conidial attachment was not significant for some Ko strains at either of the time points, a trend could be observed in the two experiments, in which knockout of *LmSNF1* resulted in an impairment in the percentage of conidia that could attach to the plant surface.

### Knockout of *LmSNF1* causes down-regulation of cell wall degrading enzyme genes

Expression of 12 CWDE genes and three pathogenicity genes was investigated in WT after incubation in CD supplemented with 1% glucose for 48 hours or in canola tissue at 4 dai. For eight of the 15 genes, expression could not be detected in glucose medium. By contrast, expression could be detected for all the 15 genes *in planta* at 4 dai. *In planta* up-regulation was evident for 14 genes (Table S3). In CD medium containing pectin, 11 CWDE genes were shown to be down-regulated in the two Ko strains compared to in WT (Table 2). Expression of one gene encoding a chitin deacetylase was at similar level in WT and Ko strains. Among the three selected pathogenicity genes, two were expressed at similar levels across the strains. The expression of the gene encoding the pathogenicity protein LopB was diminished in the Ko strains, indicating that this gene is under control of *LmSNF1*.

**Table 2:** Expression of selected cell wall degrading enzyme genes in the wild-type and two *LmSNF1* knockout strains after culturing in minimal medium supplemented with 1% pectin.

#	Genbank	Gene description	Expression relative to *ACT1*		
	Acc. Number		WT	Ko2	Ko4
1	CBX90811	Pectate lyase	2497.9±610.5 a^a^	0.4±0.1 b	Non
2	CBX92557	Pectate lyase	320.0±25.3 a	2.2±0.3 b	5.3±0.8 b
3	CBY02118	Rhamnogalacturonate lyase	1476.2±527.3 a	2.7±1.3 b	2.3±0.4 b
4	CBX99296	Pectin or pectate lyase	13.1±3.1 a	0.1±0.1 b	0.1±0.1 b
5	CBX90808	Carbohydrate esterase	11.9±3.7 a	Non	0.1±0.1 b
6	CBX91774	Chitin deacetylase	12.6±3.3 a	14.5±2.8 a	7.8±1.0 a
7	CBX92723	SGNH_hydrolase	25.1±8.2	Non	Non
8	CBX93727	SGNH_hydrolase	292.9±52.0 a	33.0±4.8 b	39.6±19.3 b
9	CBY01967	Beta-1,3-glucanase	52.6±15.3 a	0.6±0.1 b	3.3±1.2 b
10	CBY00718	Glucan 1,4-alpha-glucosidase	258.4±32.7 a	16.0±1.5 b	12.4±2.2 b
11	CBX93703	Glycoside hydrolase	123.8±26.9 a	9.7±3.6 b	5.7±1.6 b
12	CBX90249	Glycogen debranching enzyme	429.1±78.4 a	7.3±1.4 b	9.9±4.1 b
13	AAM89498	Isocitrate lyase	162.3±22.2 a	146.1±16.8 a	106.8±27.5 a
14	AAP40632	Pathogenicity protein LopB	212.0±20.5 a	14.7±1.8 b	20.5±2.9 b
15	AM933613	Plasma membrane ATPase 1	43.2±9.1 a	40.0±4.1 a	32.1±5.6 a

a Data (mean±SE) followed by the same letter do not differ based on Fisher's LSD test at *P*≤0.01 (n = 3).

### Knockout of *LmSNF1* causes loss of fungal pathogenicity

**Figure 7:**
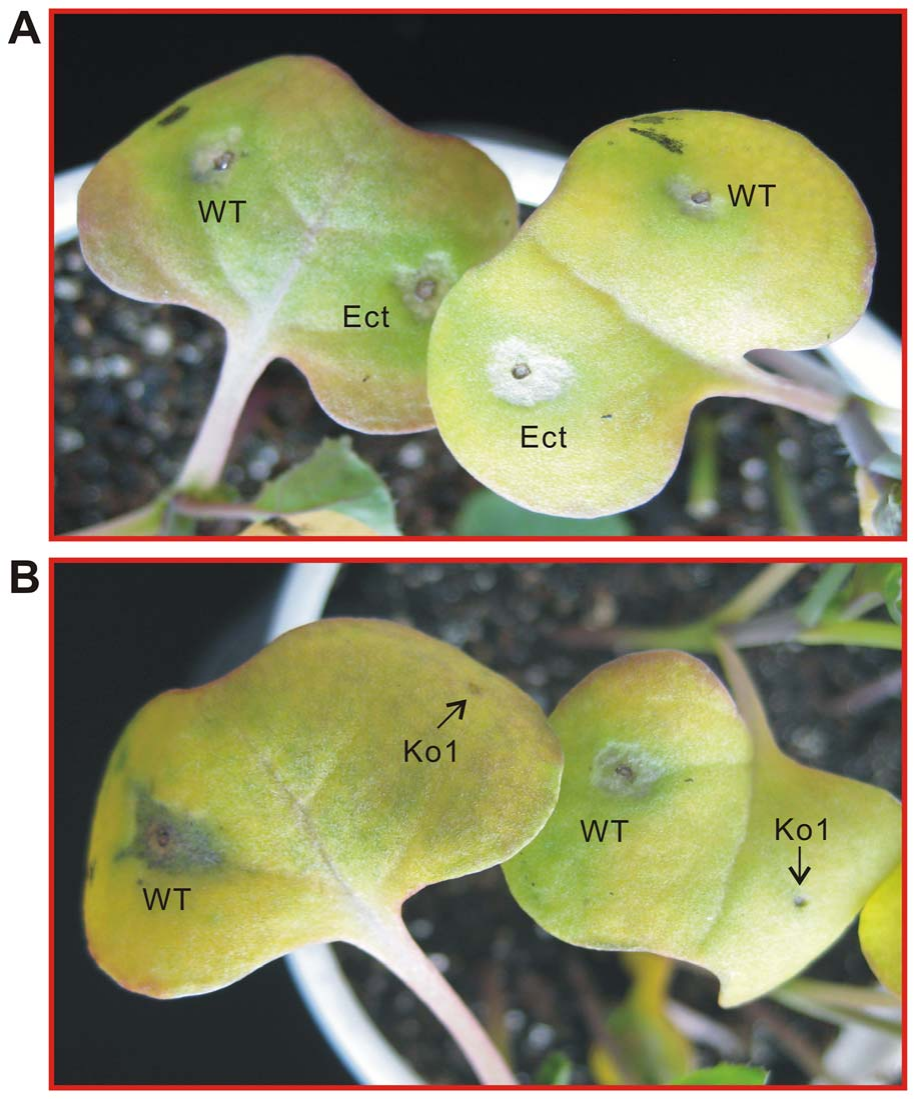
Pathogenicity of *Leptosphaeria maculans* strains. Cotyledons of canola cultivar Westar were inoculated with 5-μl droplets of conidial suspension from each strain at a concentration of 1×10^6^ conidia/ml. The pictures were taken at 6 day after inoculation.WT, the wild-type; Ect, the ectopic insertion strain; Ko1, *LmSNF1* knockout strain.

The pathogenicity of all Ko strains and Ect were assessed on cotyledons of canola. After inoculation, blackleg symptom development was monitored from 4 to 7 dai. For WT and Ect, blackleg lesions started to become evident at 4 dai and enlarged from that point on (Figure S5). At 7 dai, all cotyledons inoculated with WT and Ect strains developed typical blackleg lesions (Figure 7A). By contrast, none of the six Ko strains caused any lesions (Figure 7B). Similar results were observed after inoculations with two concentrations of conidia, 1×10^5^ or 1×10^6^ conidia/ml. This indicates that knockout of *LmSNF1* renders the fungus non-pathogenic on canola.

## Discussion

### Targeted gene knockout in *L. maculans*

Successful introduction of foreign DNA into a fungal genome is dependent on the DNA double-strand break (DSB) repair mechanisms. Eukaryotes have two main DSB repair pathways: homologous recombination and nonhomologous end-joining (NHEJ) [50]. A gene replacement event requires the occurrence of DSB in the vicinity of the targeted gene and the utilization of homologous recombination to repair the DSB. However, although some species such as *S. cerevisiae* use the homologous recombination system more frequently, many filamentous fungi seem to prefer NHEJ [51].

Targeted gene knockout has been reported to be difficult in *L. maculans*: flanking sequences greater than 7 kb are required to allow the occurrence of homologous recombination and hundreds of transformants need to be screened [41], [42]. In the present study, the first construct contained 5′ (1.1-kb) and 3′ (0.3-kb) gene-flanking sequences. Transformation using this construct produced 21 transformants and two of which were shown to be gene replacement strains. The gene replacement efficiency (2/21 = 9.5%) is comparable with that observed in other fungi when ∼1 kb flanking sequences from both the 5′ and 3′ regions were used, for example 13.0% in *F. graminearum* [6] and 14.8% in *S. nodorum* [48]. Wilson et al. [42] obtained two *L. maculans* gene replacement strains from 23 transformants by using a construct carrying 3.7-kb 5′ and 3.2-kb 3′ homologous sequences. These authors also used a construct carrying 1.4-kb 5′ and 1.5-kb 3′ homologous sequences but failed to generate any gene replacement strains, albeit 89 transformants were obtained. Based on the gene replacement efficiencies observed in the present study and by Wilson et al. [42], it can be inferred that the NHEJ DSB repair mechanism is preferentially used in *L. maculans* relative to other filamentous fungi, and that gene replacement in this species maybe not as difficult as previously reported.

In the present study, we also used a split-marker gene replacement strategy to construct the transforming DNA. Previously, it was reported that the efficiency of gene replacement in other fungi could be enhanced by employing a split-marker approach where regions flanking the gene of interest are fused to overlapping partial segments of a selectable marker [47], [52]. This was supported by the present study: five transformants were obtained and four of them were *LmSNF1* replacement strains.

### Expression of *LmSNF1* in *L. maculans*

The expression of *LmSNF1* was similar in CD medium with or without glucose. This is consistent with earlier reports on the expression of *SNF1* in *S. sclerotiorum* [18] and *F. graminearum* [20], in which the expression was independent of the presence of glucose. By contrast, the expression of *LmSNF1* was higher in pectin and V8 media than in CD and CD with glucose. This also is consistent with what was observed with respect to the expression of *SNF1* in *Colletotrichum gloeosporoides* f. sp. *malvae* [53], in which expression was consistently higher in glycerol than in glucose cultures, but differed from a report on *S. sclerotiorum* [18], in which *SNF1* expression was apparently constant in media containing either glucose or other carbon sources, including pectin. Based on the expression data obtained in the present study, it seems that some of the components found in V8 juice, including pectin, are responsible for the *LmSNF1* up-regulation.

The up-regulation of *LmSNF1* observed at 48 hai is consistent with reports of *C. gloeosporoides* f. sp. *malvae* [53] and *S. sclerotiorum* [18]. In both species, expression of *SNF1* peaked during the early stages of infection and then remained constant at much lower levels. At 48 hai, most of the *L. maculans* spores had germinated and the fungus started to invade the plant by extensive hyphal growth. Thus, it appears that *LmSNF1* expression is related to the phase of growth rather than to the physiological conditions encountered during plant invasion.

### Expression of CWDE and pathogenicity genes in the *LmSNF1* Ko strains

The 12 CWDE genes were chosen from the CAZy database, which hosts four groups of enzymes: glycoside hydrolases (GHs), glycosyltransferases (GTs), polysaccharide lyases (PLs), and carbohydrate esterases (CEs). Among these four groups, CE, GH, and PL are often considered as CWDEs due to their important roles in plant biomass decomposition by fungi and bacteria [54]. In the present study we chose four genes from each of these three groups to investigate their expression in the *LmSNF1* Ko strains.

Eleven out of these 12 genes were up-regulated during pathogenesis at 4 dai. One gene encoding a glycogen debranching enzyme had a similar expression profile in the glucose media and *in planta.* In *Blumeria graminis* f. sp. *hordei*, glycogen debranching enzymes are abundant during spore germination but become sparse in the later stages of infection [55]. While the 4 dai time point examined in this study occurred after the up-regulation stage of this gene, the glycogen debranching enzyme gene was, nonetheless, among the 11 CWDE genes that were down-regulated in the *LmSNF1* Ko strains (Table 2). The expression of one gene encoding a chitin deacetylase was at similar levels in WT and *LmSNF1* Ko strains, indicating that this gene is not under regulation of *LmSNF1*. That some CWDE genes were not down-regulated was also observed in the *SNF1* knockout strains of other plant pathogenic fungi [19], [56]. In *Ustilago maydis* in particular, knockout of *SNF1* reduced the expression of several CWDE genes but also increased the expression of two xylanase genes [56].

The three pathogenicity-related genes (isocitrate lyase, *LopB* and the plasma membrane ATPase 1) had been studied previously [32], [33], [36]. All of these genes are under control of glucose repression (Table S3) and in general, the *in vitro* and *in planta* expression of these genes was consistent with previously described expression profiles. In the *LmSNF1* Ko strains, only the *LopB* gene was down-regulated and the expression of the isocitrate lyase and the plasma membrane ATPase 1 was similar to the WT, indicating that the latter two are not under control of the *LmSNF1* derepression.

### Function of *LmSNF1* on vegetative growth, sporulation, conidial germination and attachment

The radial growth of *LmSNF1* Ko strains was similarly impaired when they were grown on media with or without glucose. This observation is consistent with reports in *M. oryzae* [19] and *P. digitatum* [22], but conflicts with all other reports, in which the *SNF1* mutants grew similarly with WT in glucose containing media. The *SNF1* genes have been reported to control sucrose and pectin utilization in differentially in various plant pathogenic fungi. For example, the growth of *SNF1* mutants of *C. carbonum* [10] and *V. dahliae* [21] was similar to the wild-types when they were grown on media with sucrose as the only carbon source, whereas the growth of *SNF1* mutants of *F. graminearum* [20] and *P. digitatum* [22] was significantly impaired. The growth of *C. carbonum* and *P. digitatum SNF1* mutants was seriously affected when these fungi were grown on media with pectin. The growth of *V. dahliae SNF1* mutants [21] and *LmSNF1* Ko strains was partially affected, whereas the growth of a *F. oxysporum SNF1* mutant [17] was not affected when it was grown on pectin-supplemented media. The differences in carbon utilization by *SNF1* mutants indicate the potential existence of distinct carbon utilization mechanisms during infection of plants by pathogenic fungi. Furthermore, in *S. cerevisiae*, *SNF1* also has been shown to be involved in the regulation of nitrogen metabolism, gluconeogenesis, and the glyoxylate and tricarboxylic acid cycles as well as in respiration and β-oxidation [57], [58]. It is possible that *LmSNF1* regulates these processes in *L. maculans* and thus affects its growth on different media.

Sporulation and spore germination were also affected by the loss of *LmSNF1*, which agreed with a report on the *F. graminearum SNF1* mutant [20]. In a *SNF1* mutant of *C. carbonum*, both sporulation and spore germination were similar to the wild-type [10]. In a *SNF1* mutant of *M. oryzae*, the sporulation ability was abolished [19], while in a *SNF1* mutant of *P. digitatum* sporulation was reduced but the spores germinated normally [22]. Sporulation and spore germination in filamentous fungi are controlled by multiple pathways and factors including nutrients, light, salt, and oxygen [59], [60]. The lower production of conidia of the *LmSNF1* Ko strains could result from inadequate or unbalanced nutrient accumulation during spore formation. *LmSNF1* also could trigger the expression of genes necessary for conidial germination. Moreover, an inability to accumulate the necessary nutrients or to trigger the genes involved in weakening the conidial cell wall architecture could delay or prevent conidial germination.

The attachment of fungal spores to the plant surface is the first step in committing a pathogen to the establishment of disease. Knockout of *LmSNF1* affected the conidial attachment to the plant surface. In plant pathogenic fungi, two basic strategies for spore attachment to the host surface have been postulated: “passive attachment” resulting from the presence of preformed materials on the fungal surface and “active attachment” requiring the *de novo* production of fungal adhesives [61]. CWDEs capable of degrading components of the plant cuticle, such as cutinase [62] and lipase [48], [63], have been demonstrated to be important for fungal attachment after a brief period in contact with the plant. Esterase, including lipase and cutinase, produced by conidia of *L. maculans* during the stages of germination might be important for active attachment. Impairment of conidial attachment in the *LmSNF1* Ko strains is likely a result of the down-regulation of esterases or other enzymes required for attachment.

### *LmSNF1* as a pathogenicity factor

In plant pathogenic fungi, *SNF1* orthologs are widely known to be required for fungal virulence, mostly by activating the expression of CWDEs. Two groups can be identified among the plant pathogenic fungal species in which the *SNF1* gene has been knocked out. The first group includes *M. oryzae* and *U. maydis.* Knockout of *M. oryzae SNF1* did not affect the expression of the tested CWDE genes but abolished the fungal pathogenicity [19], whereas knockout of *U. maydis SNF1* caused down-regulation of some but up-regulation of other CWDE genes and slightly decreased fungal pathogenicity [56]. The second group includes *P. digitatum*, *F. graminearum*, *F. oxysporum*, *V. dahlia*, *C. carbonum* and *L. maculans*, in which knockout of *SNF1* abolished the expression of most or all of the tested CWDE genes and decreased the fungal pathogenicity. Interestingly, both species in the first group are known to produce well-developed appressoria during penetration. In the second group, only *C. carbonum* and *V. dahliae* produce appressoria, but both species do not require the formation of appressoria to cause disease [64], [65]. Activation of CWDEs during the early stages of infection maybe more important in species of the second group than in those of the first group, and the link between *SNF1*, CWDEs and pathogenicity is more strictly complied.

*L. maculans* invades the plant via the stomatal apertures [41]. The hyphae need to grow on surface of the plant epidermis until they encounter a stoma. During this short period, attachment to the plant surface is critical because water can wash away deposited conidia and rain drops can cause strong air movement over short distances. It can be inferred that impairment of conidial attachment in the *LmSNF1* Ko strains would contribute to the loss of pathogenicity under natural conditions. However, the wound inoculation protocol employed in the current study bypassed the attachment and penetration phases of infection. Thus, the observed loss-of-pathogenicity most likely reflects impaired spore germination and reduced vitality of the germinated spores. Biochemically the reduced virulence of *SNF1* mutants in *C. carbonum* and *F. oxysporum* was explained by their reduced ability to utilize complex carbon sources and the reduced expression of CWDEs [10], [17]. Similar mechanisms also may explain the loss-of-pathogenicity of the *LmSNF1* Ko strains. During the initial interaction of *L. maculans* with canola, germination of the spores or the presence of chemicals on plant surface activate *LmSNF1* and result in the subsequent induction of CWDEs, which function as virulence factors that contribute to pathogenesis.

In summary, the present study demonstrated that the *LmSNF1* gene is required by *L. maculans* to cause blackleg disease on canola. The down-regulation of CWDE genes in the *LmSNF1* Ko strains provides additional evidence for the importance of CWDEs in fungal pathogenicity. In addition, the study also provided data to support the suitability of *L. maculans* for targeted gene knockout.

## Supporting Information

Figure S1 **Comparison of the fungal phylogenetic tree based on sequences of 70 protein encoding genes (left) and the tree based on** ***SNF1*** **sequences (right).** Clades identical in the two trees are connected by dotted lines.
(PDF)

Figure S2 **PCR analysis of LmSNF1 gene knockout mutants of** ***Leptosphaeria maculans*** **.** WT, the wild-type; Ect, the ectopic insertion strain; Ko, *LmSNF1* knockout strains. Labels on top indicate the target genes (forward/reverse primers).
(PDF)

Figure S3 **Growth of** ***Leptosphaeria maculans*** **strains in minimal media supplemented with 15% V8 juice (A), 1% glucose (B) or 1% pectin (C).**
(PDF)

Figure S4 **Germinated (arrow) and ungerminated (arrow head) conidia of** ***Leptosphaeria maculans*** **under microscope (10×40).**
(PDF)

Figure S5 **Lesion development on canola cotyledons after inoculated with** ***Leptosphaeria maculans*** **strains.**
(PDF)

Table S1 **Sequences of primers indicated in** **Figure 1** **.**
(PDF)

Table S2 **Primers for CWDEs and pathogenicity genes.**
(PDF)

Table S3 **Expression of selected CWDE and pathogenicity genes in** ***Leptosphaeria maculans*** **after cultured in minimal medium supplemented with 1% glucose or in infected canola cotyledons at 4 day after inoculation.**
(PDF)
