# Supplementary material for: The LmSNF1 Gene Is Required for Pathogenicity in the Canola Blackleg Pathogen Leptosphaeria maculans
Source: PLoS One. 2014 Mar 17;9(3):e92503. doi: 10.1371/journal.pone.0092503 (PMC3956939; doi:10.1371/journal.pone.0092503)
Supplement: Figure S1 — Comparison of the fungal phylogenetic tree based on sequences of 70 protein encoding genes (left) and the tree based on SNF1 sequences (right). Clades identical in the two trees are connected by dotted lines. (PDF) [file pone.0092503.s001.pdf]

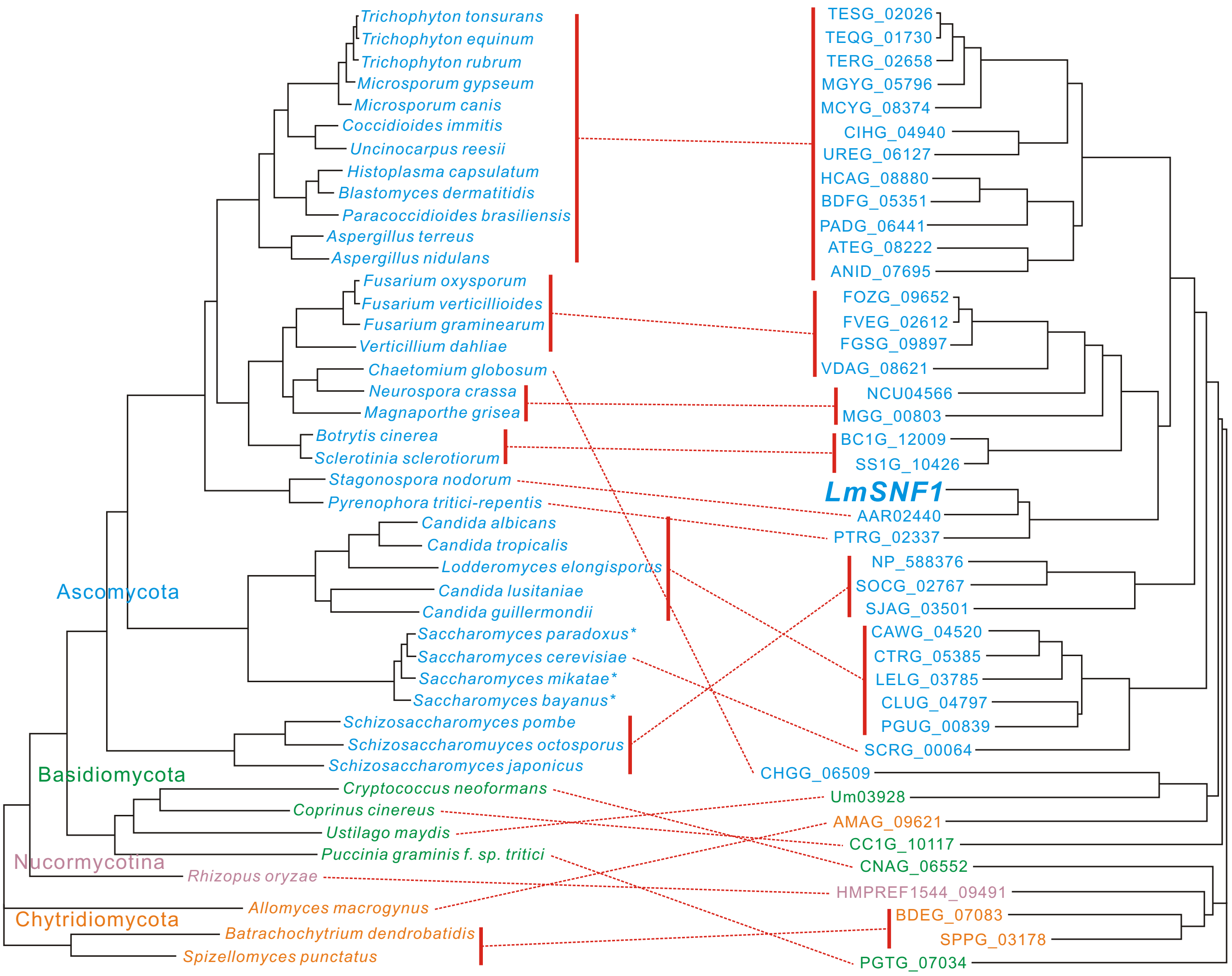

Figure S1. Comparison of the fungal phylogenetic tree based on sequences of 70 protein encoding genes (left) and the tree based on *SNF1* sequences (right). Clades identical in the two trees are connected by dotted lines.
