# Supplementary material for: The LmSNF1 Gene Is Required for Pathogenicity in the Canola Blackleg Pathogen Leptosphaeria maculans
Source: PLoS One. 2014 Mar 17;9(3):e92503. doi: 10.1371/journal.pone.0092503 (PMC3956939; doi:10.1371/journal.pone.0092503)
Supplement: Figure S2 — PCR analysis of LmSNF1 gene knockout mutants of Leptosphaeria maculans . WT, the wild-type; Ect, the ectopic insertion strain; Ko, LmSNF1 knockout strains. Labels on top indicate the target genes (forward/reverse primers). (PDF) [file pone.0092503.s002.pdf]

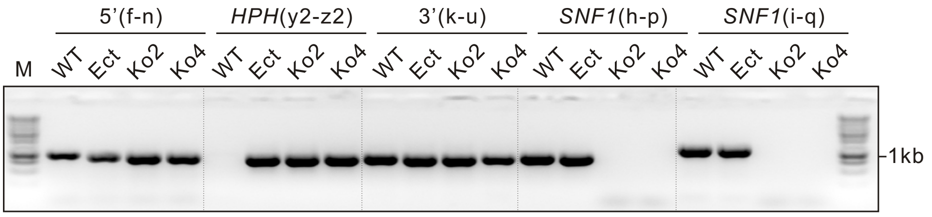

Figure S2. PCR analysis of *LmSNF1* gene knockout mutants of *Leptosphaeria maculans*. WT, the wild-type; Ect, the ectopic insertion strain; Ko, *LmSNF1* knockout strains. Labels on top indicate the target genes (forward/reverse primers).
