# Supplementary material for: The LmSNF1 Gene Is Required for Pathogenicity in the Canola Blackleg Pathogen Leptosphaeria maculans
Source: PLoS One. 2014 Mar 17;9(3):e92503. doi: 10.1371/journal.pone.0092503 (PMC3956939; doi:10.1371/journal.pone.0092503)
Supplement: Figure S3 — Growth of Leptosphaeria maculans strains in minimal media supplemented with 15% V8 juice (A), 1% glucose (B) or 1% pectin (C). (PDF) [file pone.0092503.s003.pdf]

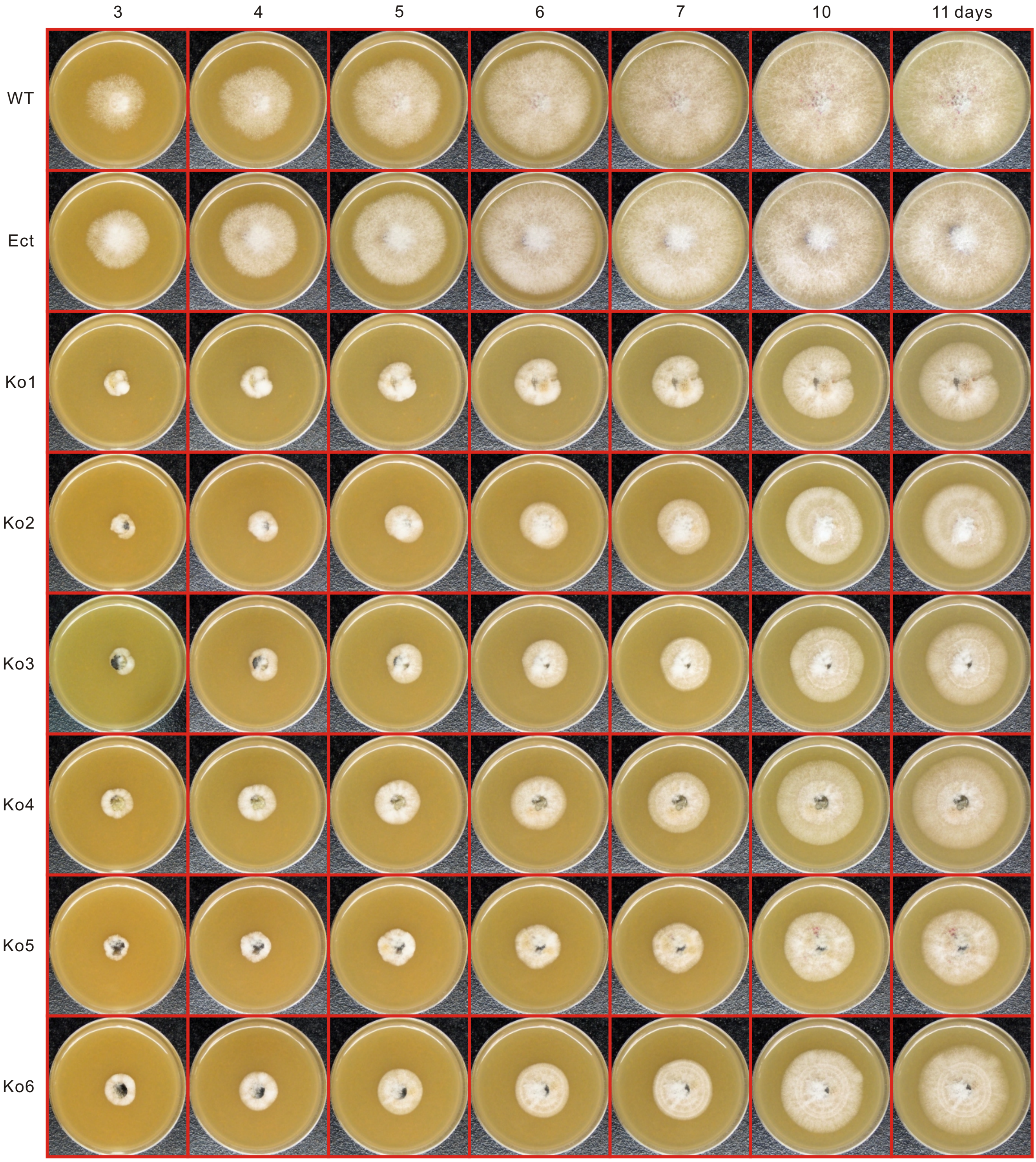

Figure S3A. Growth of *L. maculans* strains in minimal media supplemented with 15% V8 juice.

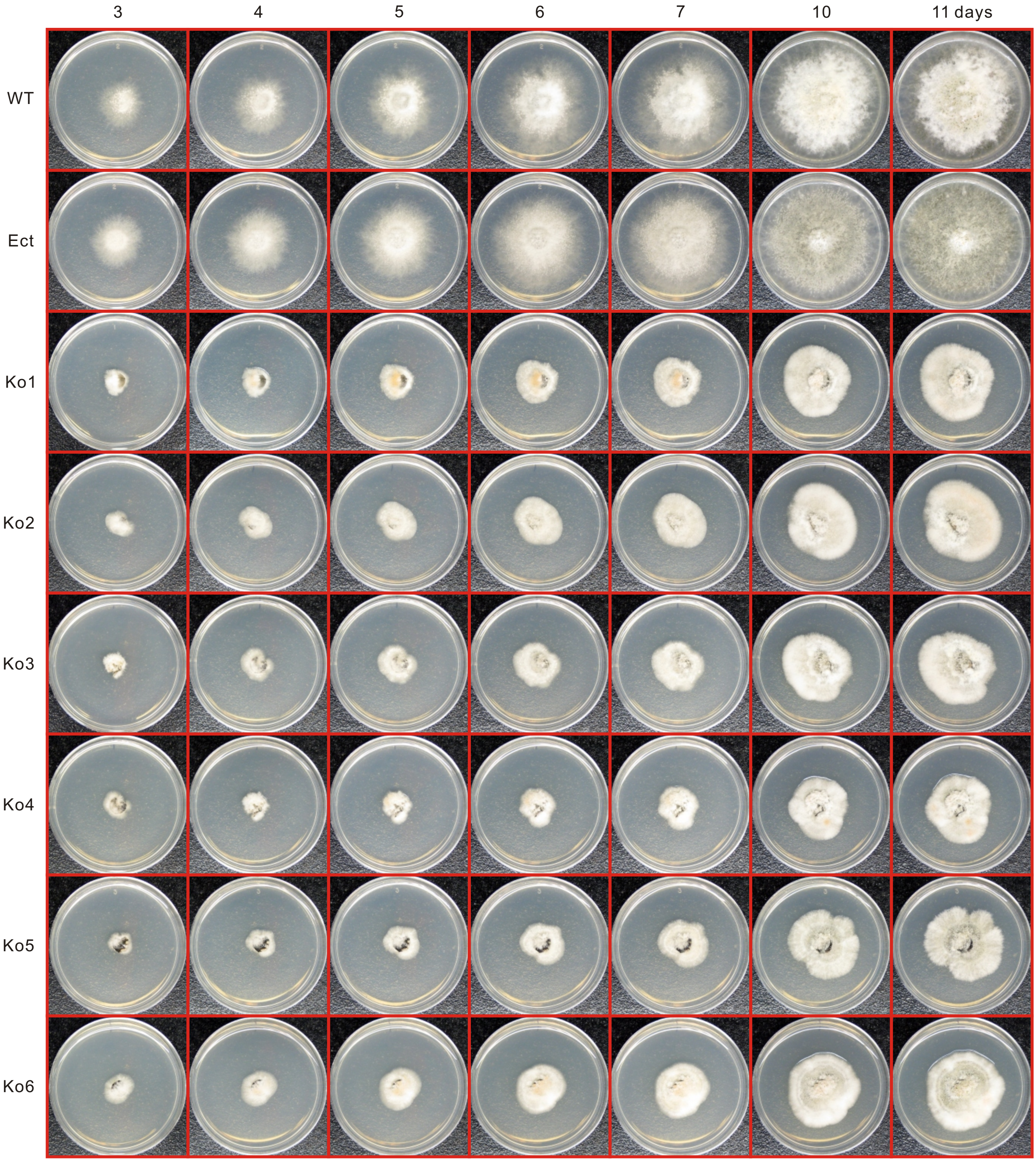

Figure S3B. Growth of *L. maculans* strains in minimal media supplemented with 1% glucose.

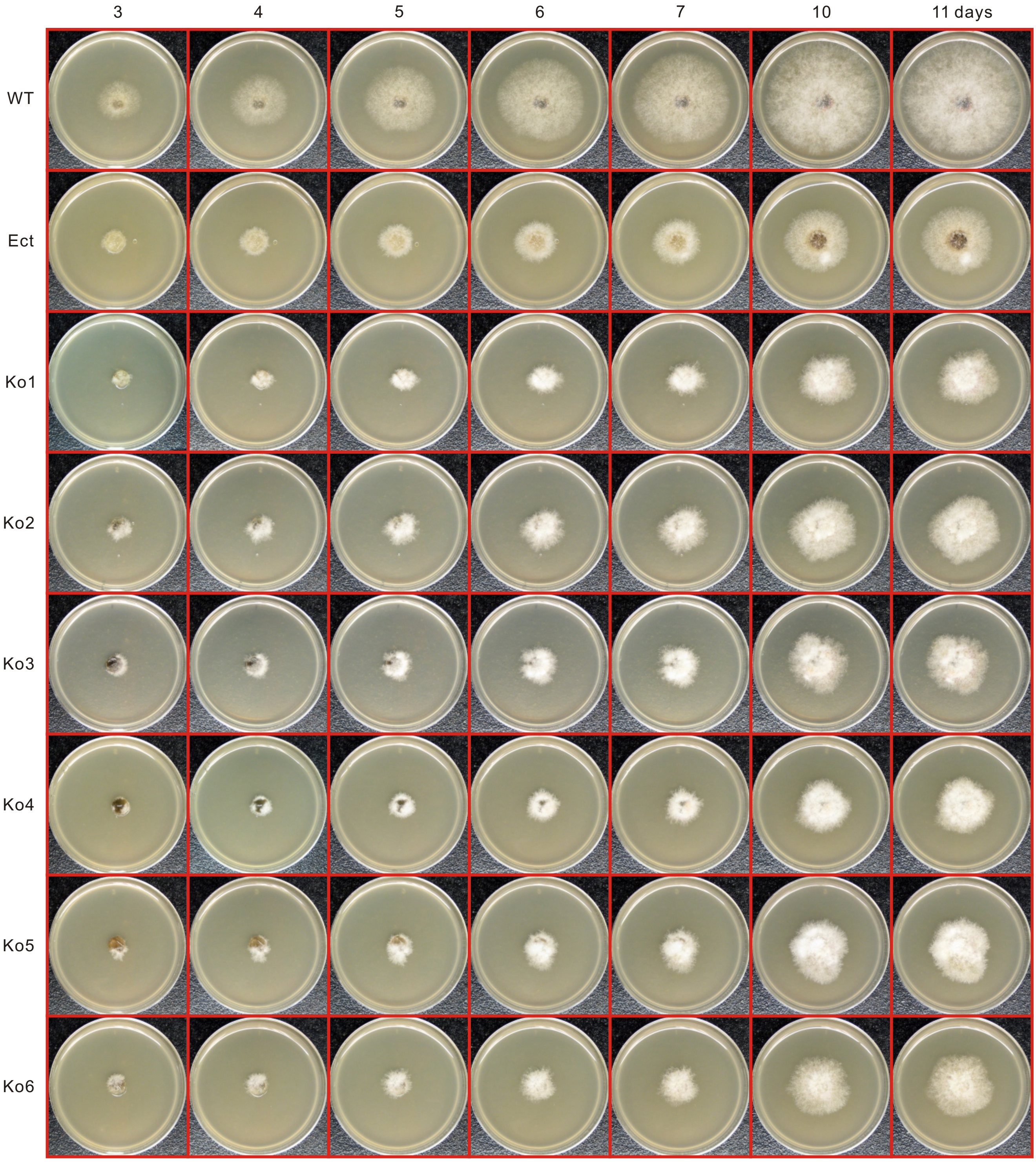

Figure S3C. Growth of *L. maculans* strains in minimal media supplemented with 1% pectin.
