# Supplementary material for: The LmSNF1 Gene Is Required for Pathogenicity in the Canola Blackleg Pathogen Leptosphaeria maculans
Source: PLoS One. 2014 Mar 17;9(3):e92503. doi: 10.1371/journal.pone.0092503 (PMC3956939; doi:10.1371/journal.pone.0092503)
Supplement: Figure S4 — Germinated (arrow) and ungerminated (arrow head) conidia of Leptosphaeria maculans under microscope (10×40). (PDF) [file pone.0092503.s004.pdf]

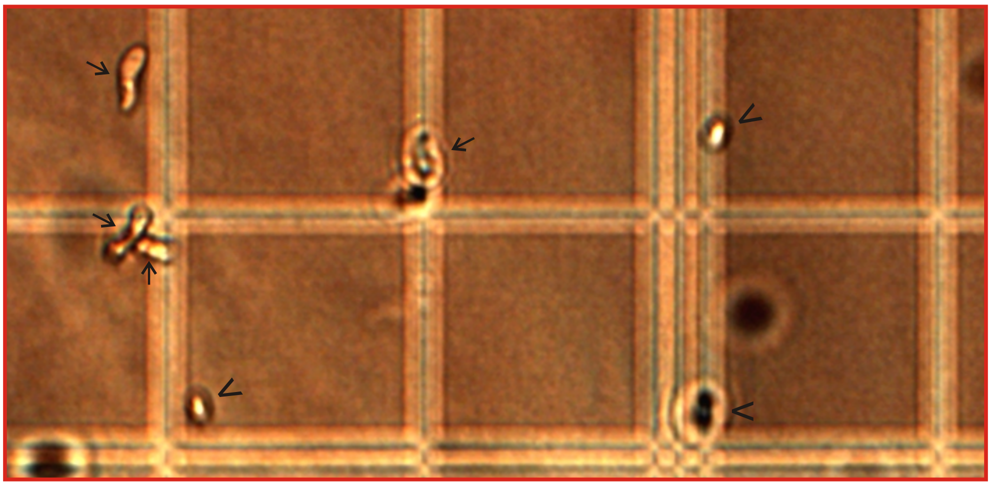

Figure S4. Germinated (arrow) and ungerminated (arrow head) conidia of *Leptosphaeria maculans* under microscope (10 × 40).
