# Supplementary figures and images for: The LmSNF1 Gene Is Required for Pathogenicity in the Canola Blackleg Pathogen Leptosphaeria maculans
Source: PLoS One. 2014 Mar 17;9(3):e92503. doi: 10.1371/journal.pone.0092503 (PMC3956939; doi:10.1371/journal.pone.0092503)

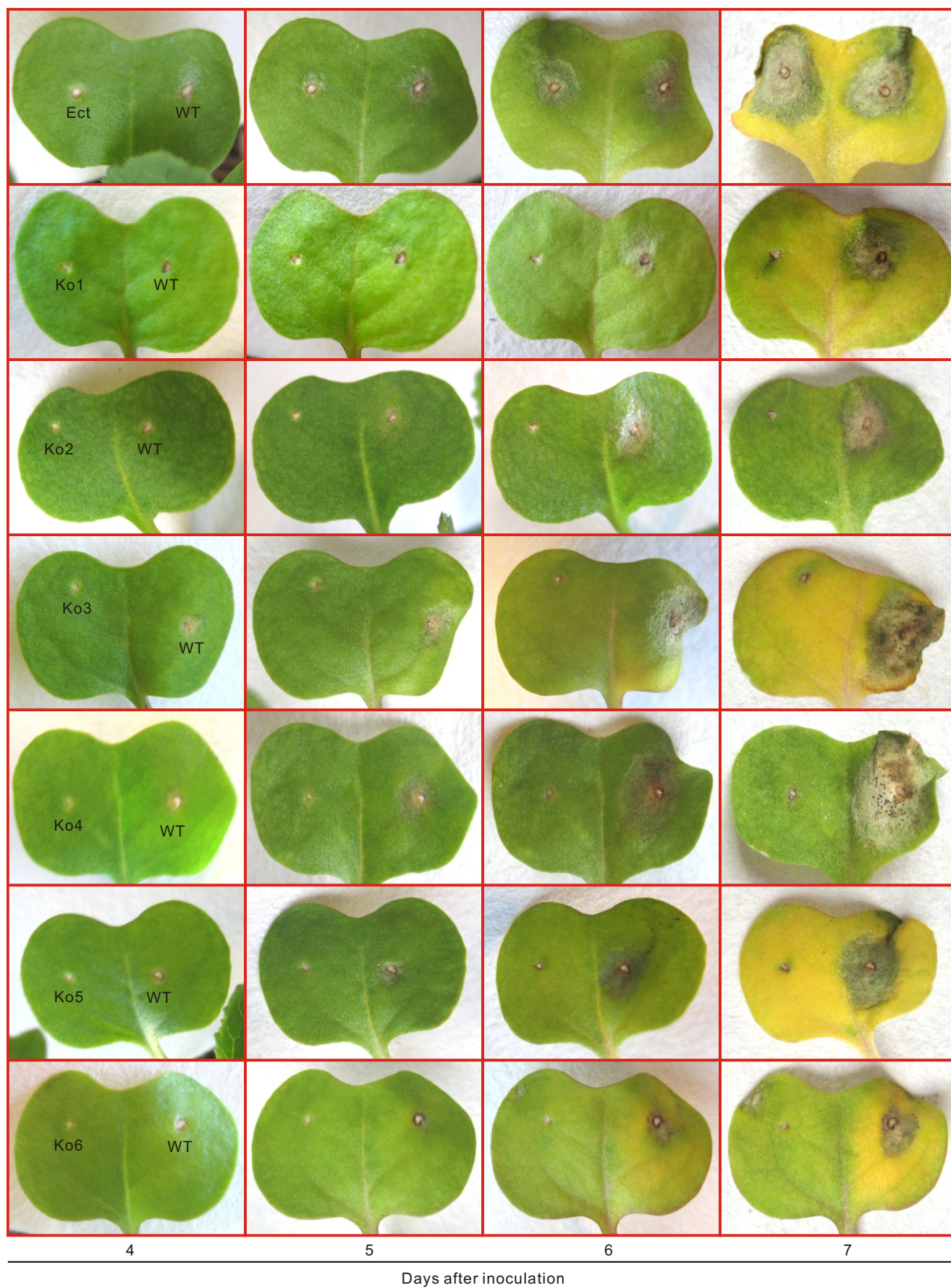

**Figure S5.** Lesion development on canola cotyledons after inoculated with *L. maculans* strains.

Supplement: Figure S5 — Lesion development on canola cotyledons after inoculated with Leptosphaeria maculans strains. (PDF) [file pone.0092503.s005.pdf]
