# Supplementary material for: The LmSNF1 Gene Is Required for Pathogenicity in the Canola Blackleg Pathogen Leptosphaeria maculans
Source: PLoS One. 2014 Mar 17;9(3):e92503. doi: 10.1371/journal.pone.0092503 (PMC3956939; doi:10.1371/journal.pone.0092503)
Supplement: Table S1 — Sequences of primers indicated in Figure 1 . (PDF) [file pone.0092503.s006.pdf]

Table S1. Sequences of primers indicated in Figure 1.

Name	Sequence	Tailled sequence
a	GCGGTTACCAATAGACGAATGCGTTGATCAG	BstEII
b	GCGGTTACCAATCGTTGAGTTGCGGTATTGC	BstEII
c	CTCTGTGGACCACGAAGGAT	
d	AACATGGATGTTGTGGCTGTGA	
e	CGTAACTGCACAATGGGAAAGT	
f	ACTGCAGTCATTGGCAATTGCC	
g	TTCTTCCAGTCGGTCCCTTATC	
h	AAACCGTCGAATTCTCTCCA	
i	GATTGCAGCAGGCAACTACA	
j	GAAAGCTTGGATCCCCGGGTTTTCACTCTTCCAGTGAGGAGCAA	R-C of z3
k	GAAAGCTTGGATCCCCGGGTTTTGGTTCGTTGGATGAGGTATAG	R-C of z3
l	ATATTCAACTCGGCGTCACC	
m	CAGTCAATTGTGGGTTGACG	
n	ATGGCCCACGCCAAGATCTGGGGACGACTAGCGACTTGGAACA	R-C of y1
o	ATGGCCCACGCCAAGATCTGGGTGGATTTGGGCTGTCAGATAAG	R-C of y1
p	GCGGGCAAATCTTTAGTGAA	
q	GTTGAAGAGGGGTGTGCAAT	
r	ATACTCCTCATACCCATGCGATG	
s	CGACTAGTGTACACAGCTACCGATGACCTT	SpeI
t	CGACTAGTTATAGCACATGACTATCCAGGC	SpeI
u	TACACAACGCCCTTGTTCTC	
v	CGGCTTCTTGGTACTTTTGC	
w	GCGGTTACCCAAACATACGTACAGCACAGCA	BstEII
x	GCGGTTACCATGTGTTGTCACAGTCAAACCA	BstEII
y1	CCCAGATCTTGGCGTGGGCCATTGTGCATGG	BglII
y2	GCGGTTACCATTGTCCGTCAGGACATTGTTG	BstEII
y3	GCGGTTACCCAAGACCAATGCGGAGCATATA	BstEII
z1	GCGGTTACCAAGAATCTCGTGCTTTCAGCTT	BstEII
z2	GCGGTTACCAGATCGTTATGTTTATCGGCAC	BstEII
z3	AAACCCGGGGATCCAAGCTTTCGAGTGGAGAT	XmaI
