# Supplementary material for: The LmSNF1 Gene Is Required for Pathogenicity in the Canola Blackleg Pathogen Leptosphaeria maculans
Source: PLoS One. 2014 Mar 17;9(3):e92503. doi: 10.1371/journal.pone.0092503 (PMC3956939; doi:10.1371/journal.pone.0092503)
Supplement: Table S2 — Primers for CWDEs and pathogenicity genes. (PDF) [file pone.0092503.s007.pdf]

Table S2. Primers for CWDEs and pathogenicity genes.

Name	Forward primer	Reverse primer	Expected size (bp)
CBX90811	CAACTACGGGGGCTTGAATA	CTCTGCAGCTCAGCAATCAG	159
CBX92557	CAGGCGTGTTTCTGGAACTT	TTGAGGGTACCACCGTTCTC	126
CBY02118	TGGCCATACAAGTGGTTTGA	CTGGCCTTGGTCGAGAGTAG	153
CBX99296	TGTCTATACGCCCAACACCA	CAGGATGCAGTGACAGAGGA	159
CBX90808	GACTCATCATCAGCCCCATT	GCTACATCGAGGGAGCTACG	133
CBX91774	TGCACTAGCCTACGACGATG	GAGCGGCTTTTTGGTTGTAG	134
CBX92723	CCAGCCAAGAACATCTCCAT	CAGCCAGATTCTCGTTCACA	154
CBX93727	CAATCCAAACATGCAAATCG	TTATGCGTGTACGTGGCAAT	120
CBY01967	CGAGGCTGGTAATTTCGGTA	TCAGCAAGATACTGCGGTTG	140
CBY00718	GGAGGACGGTTATTGGAGGT	CGAGCAATTCGACAAGAACA	141
CBX93703	AGAAACACCCGTCACCTTTG	GGAATTTTGCAGCCACAAGT	160
CBX90249	CCAGGAAGCTCTTCAACGTC	GATTCCCACCGAAGATCAGA	146
AAM89498	GGACACATGGCTGGAAAAGT	CGTGGGTCGATTGTAGAGGT	158
AAP40632	CCAGATCCGCATCTACCTGT	CCGCAATTTCATGGAGAGAT	100
AM933613	TGACTGGTGACGGTGTCAAT	ATCTGGCGAGCAGTCTTGAT	160
