# Supplementary material for: The LmSNF1 Gene Is Required for Pathogenicity in the Canola Blackleg Pathogen Leptosphaeria maculans
Source: PLoS One. 2014 Mar 17;9(3):e92503. doi: 10.1371/journal.pone.0092503 (PMC3956939; doi:10.1371/journal.pone.0092503)
Supplement: Table S3 — Expression of selected CWDE and pathogenicity genes in Leptosphaeria maculans after cultured in minimal medium supplemented with 1% glucose or in infected canola cotyledons at 4 day after inoculation. (PDF) [file pone.0092503.s008.pdf]

Table S3. Expression of selected CWDE and pathogenicity genes in *Leptosphaeria maculans* after cultured in minimal medium supplemented with 1% glucose or in infected canola cotyledons at 4 day after inoculation.

#	GenBank Number	Gene description	Expression relative to *ACT1*			*In Planta*
			Glucose	4 dai	P value[a]	Activation
1	CBX90811	Pectate lyase	No	0.9±0.2	NA	Y
2	CBX92557	Pectate lyase	0.3±0.1	1.7±0.3	0.004	Y
3	CBY02118	Rhamnogalacturonate lyase	No	2.8±0.7	NA	Y
4	CBX99296	Pectin or pectate lyase	0.4±0.1	2.4±0.1	0.002	Y
5	CBX90808	Carbohydrate esterase	No	1.8±0.9	NA	Y
6	CBX91774	Chitin deacetylase	0.8±0.4	17.1±3.4	0.007	Y
7	CBX92723	SGNH hydrolase	No	0.8±0.1	NA	Y
8	CBX93727	SGNH hydrolase	0.2±0.1	5.9±0.4	0.003	Y
9	CBY01967	Beta-1,3-glucanase	No	8.8±1.3	NA	Y
10	CBY00718	Glucosidase	No	7.9±1.9	NA	Y
11	CBX93703	Glycoside hydrolase	No	4.9±1.8	NA	Y
12	CBX90249	Glycogen debranching	1.1±0.2	2.0±0.6	0.178	N
13	AAM89498	Isocitrate lyase	No	43.0±10.1	NA	Y
14	AAP40632	Pathogenicity protein LopB	0.1±0.1	3.5±1.1	0.003	Y
15	AM933613	Plasma membrane ATPase	1.4±0.2	48.3±7.8	0.009	Y

[a] P value of F test (n=3).
